# Feasibility of Intensive Chemotherapy in Hereditary Spherocytosis

**DOI:** 10.3390/hematolrep17020011

**Published:** 2025-02-24

**Authors:** Carrai Valentina, Giubbilei Cristina, Ciceri Manuel, D’Angelo Simona, Nassi Luca, Sordi Benedetta, Vannucchi Alessandro Maria, Puccini Benedetta

**Affiliations:** 1 Hematology Unit, Careggi University Hospital, 50134 Florence, Italy; carraiv@aou-careggi.toscana.it (C.V.); manuel.ciceri@unifi.it (C.M.); simona.dangelo@unifi.it (D.S.); nassil@aou-careggi.toscana.it (N.L.); benedetta.sordi@unifi.it (S.B.); a.vannucchi@unifi.it (V.A.M.); puccinib@aou-careggi.toscana.it (P.B.); 2 Center for Research and Innovation in Myeloproliferative Neoplasms, Hematology Unit, Careggi University Hospital, University of Florence, 50134 Florence, Italy

**Keywords:** hereditary spherocytosis, diffuse large B cell lymphoma, chemotherapy, rare disorders

## Abstract

Background: This study presents a young man with hereditary spherocytosis (HS) who underwent intensive chemotherapy for newly diagnosed diffuse large B-cell lymphoma (DLBCL) and achieved complete remission. This case challenges the idea of HS as a barrier to standard DLBCL treatment. Discussion: By meticulously monitoring blood counts and providing timely transfusions, the team successfully mitigated potential complications associated with chemotherapy-induced stress on red blood cells. Conclusions: This experience underscores the importance of a multidisciplinary approach and tailored treatment plans for patients with co-existing conditions, suggesting that HS should not automatically disqualify them from potentially curative therapies for aggressive lymphomas.

## 1. Introduction

We present a case of a 24-year-old male with hereditary spherocytosis (HS) who successfully underwent intensive chemotherapy for newly diagnosed diffuse large B-cell lymphoma (DLBCL). The purpose of this case report is to demonstrate the feasibility and challenges of administering intensive chemotherapy to patients with HS and DLBCL, and to highlight the importance of tailored supportive care in the management of such complex cases.

HS is an inherited hemolytic anemia caused by various genetic abnormalities affecting the RBC membrane and cytoskeleton. The most common defects involve quantitative deficiencies of alpha/beta-spectrin, ankyrin, and erythrocyte membrane protein band 4.2 and band 3 (B3p), leading to the loss of adequate intracytoplasmic vertical linkages and deformability [1].

Clinical manifestations range from mild to severe, with chronic hemolosis leading to anemia, jaundice, and splenomegaly. HS can be classified as mild, moderate, or severe based on clinical presentation and laboratory findings [2]. HS diagnosis is confirmed by erythrocyte membrane antigen (EMA) binding, osmotic fragility, and osmotic gradient ektacytometry (OGE) [3].

Splenectomy is the definitive treatment [4], but supportive measures like folic acid supplementation and RBC transfusions are crucial [2].

## 2. Clinical Case

Our patient was diagnosed with HS at age eight based on clinical features and laboratory findings. Baseline patient laboratory reports met criteria for mild disease according to Eber et al. [5] with hemoglobin 11.1 g/dL, bilirubin 1.8 mg/dL, and reticulocytes 3%.

His past medical history was significant for only two hospitalizations: a cholecystectomy at age 13 and parvovirus B19 infection at 15. He only required transfusions during the latter due to significant anemia.

**Figure 1:**
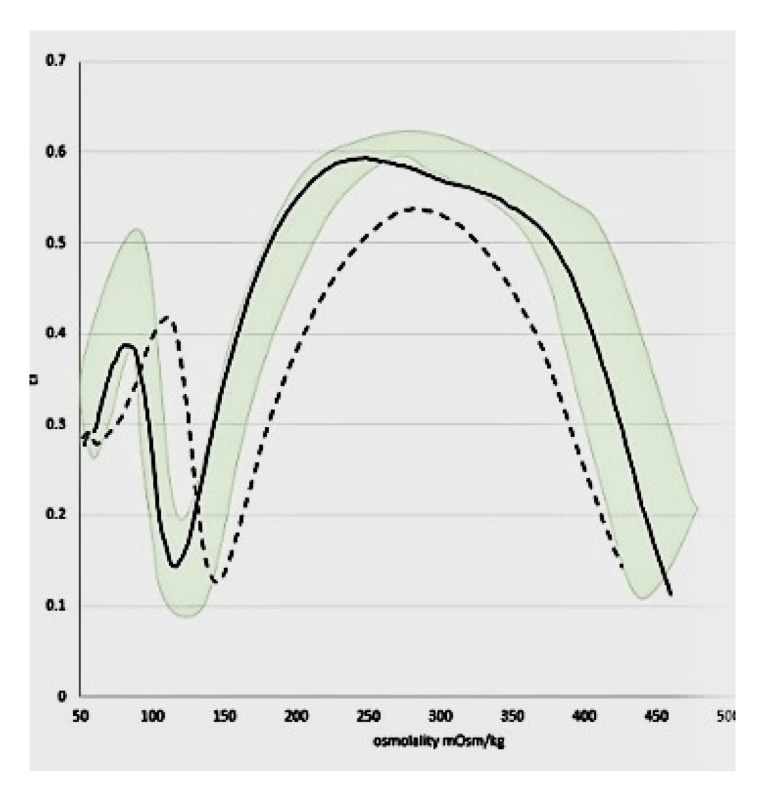
Patient’s ektacytometry pattern showing typical red blood cell deformability with downward shift.

He maintained a mild disease course managed conservatively with folic acid only. In consideration of a non-Hodgkin lymphoma diagnosis, confirmatory studies were performed [3]. Analysis revealed decreased erythrocyte osmotic resistance and a positive EMA binding test. Ektacytometry confirmed a characteristic pattern of spherocytosis in red blood cell deformability (Figure 1). This finding was further supported by protein analysis of the red blood cell membrane, which revealed a deficiency in spectrin (More Details available in Supplementary Materials).

Prior to the NHL diagnosis, laboratory results consistently indicated moderate spherocytosis. Interestingly, these values have shown a gradual, mild increase over time (Table 1).

In August 2022 when he was 23 years old, following a new onset of neurological symptoms such as occipital headache with significant neck pain associated with paresis of the tongue and difficulty speaking and chewing, a head computed tomography (CT) and magnetic resonance imaging (MRI) were performed confirming the presence of an expansive lesion with bony and extraosseous development located in the left and upper hemiportion of the clivus, involving the posterior clinoid processes of the sphenoid and the left occipital condyle: these findings raised suspicion for a lymphoproliferative disease. The solid tissue insinuated the sella turcica, imprinting the neurohypophysis and extended up to the posterior wall of the sphenoid sinus following the course of the great wing of the sphenoid, partly in the context of the left hypoglossal canal. A trans-sphenoidal biopsy was performed revealing histologic features suggestive of diffuse large B-cell lymphoma (CD20+, CD5-, CD10-, bc16+, MUM1-, bcI2-, c-Myc < 5%, EBV-, KI67 50–60%, GCB, and primitive bone).

Staging workup excluded further involvement with a bone marrow biopsy showing no lymphoma involvement; patient was therefore classified as DLBCL stage IV (bone), age adjusted (aa)IPI (International Prognostic Index) 2 (stage and LDH: lactate dehydrogenase) ECOG 0.

The multidisciplinary team established a treatment plan of six R-CHOP-21 doses (21-day cycle: rituximab 375 mg/mq, cyclophosphamide 750 mg/mq, doxorubicin 50 mg/mq, vincristine 1.4 mg/mq, and prednisone 100 mg) with four diagnostic-therapeutic lumbar punctures followed by two further cycles of HD-MTX (high-dose methotrexate 3 g/mq). We used G-CSF for primary prophylaxis [6].

At the restaging after immuno-chemotherapy and lumbar puncture, cranial MRI showed a reduction in the signal alteration involving the left occipital condyle, the left clivus and the petrous part. The fluorodeoxyglucose positron emission tomography (FDG-PET) showed a complete response to therapy (Deuville score 2); therefore, the patient performed 2 final cycles of IV HD-MTX with subsequent mandatory folin rescue [7,8].

**Figure 2:**
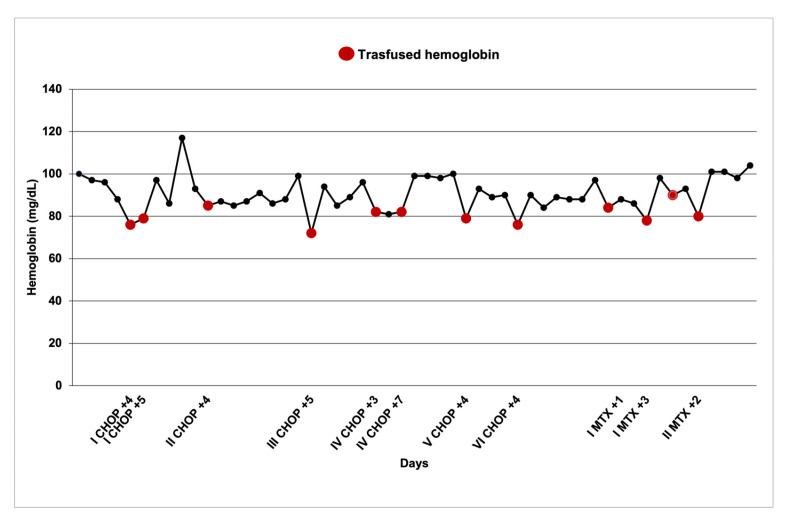
Hemoglobin nadirs coinciding with RBC transfusions. On *x*-axis, chemotherapy cycles are reported: Roman numerals indicate cycles (e.g., I, II, etc.), while Arabic numerals represent days from end of cycle (+2, +4, etc.). CHOP: Cyclophosphamide, doxorubicin, vincristine, prednisone; MTX: Methotrexate.

Given the possibility of the worsening of HS with chemotherapy-induced stress, a proactive approach was conducted. Close monitoring of hematological parameters was implemented to predict potential aplastic crises and guide timely transfusional support. Compared to the general population, our patient exhibited an earlier hemoglobin nadir (median: 4 days) with a nadir median hemoglobin of 7.85 gdL (range of 7.2–8.5 g/dL). Erythropoietin was not used due to a lack of supporting data in this specific setting especially in adults [8]. He received a total of 12 transfusions coinciding with hemoglobin nadirs (Figure 2).

The patient successfully completed all chemotherapy cycles without infections and achieved a complete remission (CR) on restaging with CT, FDG-PET and brain MRI.

In September 2024, during the last follow-up, the patient was still in CR without late toxicities.

## 3. Discussion

In patients with HS, hemolytic crises requiring supportive therapy are relatively common, particularly during periods of physical or psychological stress. These crises can vary in severity, ranging from mild to moderate episodes to more severe cases that necessitate hospital monitoring and treatment. One of the main causes of severe crises is parvovirus B19 infection, which triggers an aplastic crisis due to its erythrotropic nature and ability to suppress erythropoiesis in the bone marrow [9]. Additionally, although less frequently observed, nutritional or functional folate deficiency can lead to severe crises, referred to as megaloblastic crises [9].

Antineoplastic agents are well recognised for their effects on hematopoiesis, either through direct suppression of hematopoietic lineages or, depending on the specific agent, through cellular toxicity. This effect is further amplified in intensive chemotherapy regimens involving multiple agents [10]. Given the expected hematological toxicity, including anaemia, neutropenia, and thrombocytopenia, close monitoring is essential to provide timely supportive therapy when required.

In patients with HS, the use of multi-agent chemotherapy regimens can induce myelosuppression, leading to consequences for erythropoiesis similar to those seen in parvovirus B19-related aplastic crises. Moreover, the addition of a folate mimetic antimetabolite agent [11] further exacerbates erythroid suppression, compounding the overall hematological impact.

There are limited cases in the literature of patients with concomitant hematological malignancies and spherocytosis successfully treated using intensive standard-of-care regimens. In one reported case, the use of a multi-drug chemotherapy regimen containing vincristine was associated with greater erythroid suppression and an increased transfusion requirement compared to the average observed in paediatric patients with T-lymphoblastic lymphoma [12]. Other reports describe the coexistence of hematological malignancies with HS, where intensive treatment was not undertaken [13]. However, even the use of lower-intensity chemotherapy in these cases led to transfusion requirements.

Given the lack of data in the literature and the absence of specific guidelines for managing the coexisting conditions observed in our patient, we considered a pre-emptive approach to be beneficial. This strategy aimed to prevent hemoglobin nadirs, thereby supporting the patient throughout the entire treatment course and ensuring that chemotherapy schedules remained uninterrupted due to delays caused by insufficient hematological recovery.

Although we chose R-CHOP with close monitoring and supportive care, other approaches in patients with worse performance status and/or older age may include reduced-dose chemotherapy regimens which may have less impact on red blood cell stability in patients with HS.

This case shows that with careful monitoring and aggressive supportive measures, patients with HS can safely receive intensive chemotherapy for aggressive lymphomas. This approach may inform future management strategies for similar patients and expand treatment options for patients previously considered too high-risk for standard treatments.

## 4. Conclusions

Our experience suggests that HS should not necessarily be considered a contraindication to intensive chemotherapy. With careful monitoring and appropriate supportive measures, such as blood transfusions, these patients can potentially achieve successful outcomes with standard-of-care therapy for aggressive lymphomas. To our knowledge, this is the first reported case of the coexistence of two such rare conditions where the successful management of supportive therapy has been thoroughly described. This approach highlights the importance of a multidisciplinary team effort and a tailored treatment plan for patients with coexisting malignant and non-malignant conditions.

Prospective studies are needed to further evaluate the safety and efficacy of different chemotherapy regimens in patients with HS and hematologic malignancies. Additionally, studies to optimize supportive care protocols for these patients may improve outcomes and reduce treatment-related complications

## Figures and Tables

**Table 1:** Patient values over year with median.

Year	Hemoglobin (g/dL)	Reticulocyte (%)	Bilirubin (mg/dL)	Bipolar Spleen Diameter	Classification
2018	12.4	NA	4.3	17 cm	mild/moderate
2019	11.3	NA	4.7	17 cm	mild/moderate
2020	11.8	NA	5.4	16 cm	mild/moderate
2021	10.1	16	4.8	17 cm	moderate
2022	11.7	10	4	18 cm	moderate
Median	**11.7 (10.1–12.4)**	**13 (10–16)**	**4.7 (4–5.4)**	**17 (16–18)**	

## Data Availability

The data supporting the conclusions of this article will be made available by the authors upon reasonable request.

## Supplementary Materials

The following supporting information can be downloaded at: https://www.mdpi.com/article/10.3390/hematolrep17020011/s1, Table S1. Erythrocyte Osmotic Resistance Report; Table S2. Flow Cytometric Analysis of Red Blood Cells Stained with Eosin-5-Maleimide (EMA Binding); Table S3. Quantitative Analysis of Membrane Proteins.
