# Supplementary material for: Feasibility of Intensive Chemotherapy in Hereditary Spherocytosis
Source: Hematol Rep. 2025 Feb 24;17(2):11. doi: 10.3390/hematolrep17020011 (PMC11932310; doi:10.3390/hematolrep17020011)
Supplement: Supplementary file 1 [file hematolrep-17-00011-s001.zip › hematolrep-3380000-supplementary.pdf]

Table S1. Erythrocyte Osmotic Resistance Report:

-Glycerol Lysis Test (seconds)		reference range
pH 7.4	30	23 - 45
pH 6.85	423	> 900
**-Osmotic Resistance in NaCl Solution**		
Fresh sample	decreased	normal
after incubation	decreased	normal
-Pink test (%):	45.0	11 – 33

Table S2. Flow Cytometric Analysis of Red Blood Cells Stained with Eosin-5-Maleimide (EMA Binding)

% Reduction in Median Channel Fluorescence (MCF)	23 %	< 11 %

Table S3. Quantitative Analysis of Membrane Proteins

		Reference Range
**- SDS-PAGE Analysis (Fairbanks Method)**		
spectrin / band 3	0.96	0.98 – 1.29
ankyrin / band 3	0.18	0.11 – 0.20
total band 4/ banda 3	0.25	0.20 – 0.26
spectrin / band 4	3.79	4.05 – 5.86
ankyrin / band 4	0.73	0.43 – 0.82
**- SDS-PAGE Analysis (Laemmli Method)**		
protein 4.1 / banda 3	0.20	0.12 – 0.24
protein 4.2 / banda 3	0.17	0.13 – 0.21
protein 4.1 / protein 4.2	1.18	0.87 – 1.30
band 7 / band 3	0.16	0.10 – 0.21
